# Bilateral antrochoanal polyp: a case report of an extremely rare entity managed conservatively with a review from the past 26 years

**DOI:** 10.1097/MS9.0000000000000290

**Published:** 2023-03-24

**Authors:** Firas K. Almarri, Abdulrahman AlHumaizi, Alanoud M. Alomair

**Affiliations:** a Department of Otorhinolaryngology – Head and Neck Surgery, Ad Diriyah Hospital, Riyadh Third Health Cluster, Ministry of Health; b Department of Otolaryngology – Head and Neck Surgery, King Abdullah bin Abdulaziz University Hospital (KAAUH); c College of Medicine, Princess Nourah Bint Abdulrahman University (PNU), Riyadh, Saudi Arabia

**Keywords:** antrochoanal polyp, antral cyst, bilateral antrochoanal polyp, medical treatment, nasal obstruction

## Abstract

Antrochoanal polyp (ACP) is a well-known benign sinonasal polyp first described in the early 20th century. ACP mostly presents as a unilateral mass and is treated solely with surgical excision.

**Case presentation:**

We report a rare case of a middle-aged man presenting with nasal obstruction, rhinorrhea, and sleeping disturbances, eventually diagnosed with bilateral ACPs. After confirming the diagnosis with imaging and biopsy studies, the patient was treated conservatively, with marked improvements in his symptoms during 2–3 months of regular follow-ups. A review of the relevant literature regarding the presentation, diagnosis, and outcome of this rare entity is presented, highlighting its controversial etiopathogenesis.

**Clinical discussion:**

Presenting symptoms of ACP in most cases is unilateral progressive nasal obstruction. The occurrence of ACP bilaterally is rarely encountered in clinical practice. Diagnosis is mainly clinical and is achievable via nasal endoscopic examination and supported by computed tomography imaging. Treatment remains to be surgical, with 2 years of regular follow-ups being advised to detect any recurrence.

**Conclusion:**

This case report adds to the scarce data pool on bilateral ACPs and highlights the necessity of prudent and timely diagnosis of this uncommon entity to avoid unnecessary investigations and lengthy medical or surgical treatment. Additionally, a trial of medical therapy may provide symptomatic relief for patients who do not qualify for surgery.

Highlights

- Antrochoanal polyp (ACP) is a benign solitary lesion that originates in the maxillary sinus and may occur in adults and children.
- The etiology of ACP remains speculative, and their bilateral occurrence is extremely rare.
- ACP harbor ambiguous clinical and radiological features, entailing a considerable high index of suspicion to provide a timely diagnosis.
- Successful ACP treatment depends on complete surgical excision to eliminate the risk of recurrence.
- A frugal approach through a trial of medical treatment may provide symptomatic relief in patients who are otherwise unfit for surgery.

## Introduction

Antrochoanal polyp (ACP) is a benign lesion arising from the mucosa of the maxillary sinus and extending through the natural or accessory ostium into the middle meatus, protruding posteriorly through the choanae into the nasopharynx (NP)^1^. The etiology of ACP remains speculative, and its incidence is more common in children than adults^2^,^3^. ACP generally manifests as but is not limited to progressive unilateral nasal obstruction and discharge^1^. These symptoms may overlap with other pathologies, creating a diagnostic dilemma. ACPs are mostly unilateral, and their bilateral occurrence is extremely rare, with few recorded cases in the literature^4^–^14^. Herein, we report a rare and unusual case of bilateral ACPs managed conservatively, intending to contribute to the scarce data pool by emphasizing its presentation, diagnosis, treatment, and outcome. We also reviewed pertinent literature related to this rare entity while highlighting its vague etiopathogenesis. This case report has been reported in line with the SCARE (Surgical CAse REport) 2020 criteria^15^.

## Case report

**Figure 1:**
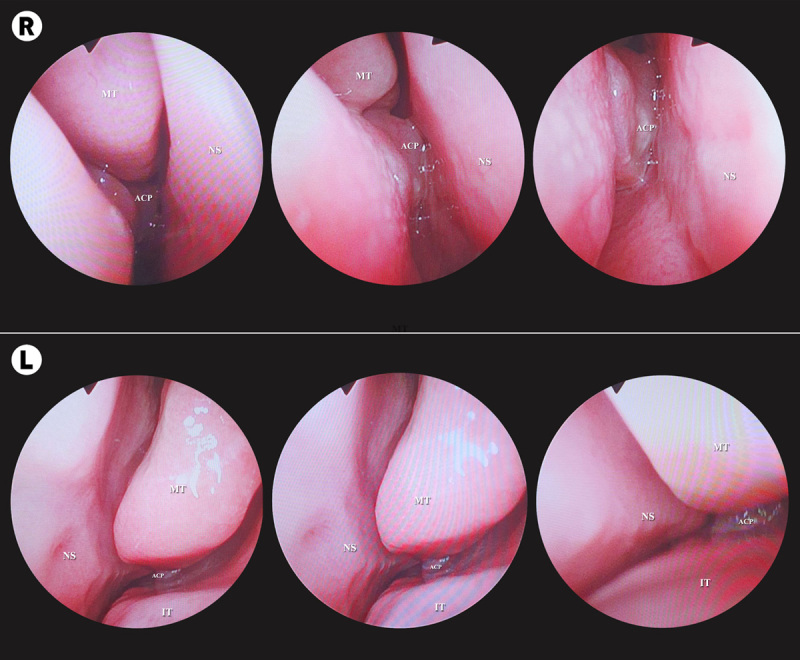
Endoscopic view of an antrochoanal polyp in the right (R) and left (L) nasal cavity. ACP, antrochoanal polyp; IT, inferior turbinate; MT, middle turbinate; NS, nasal septum.

**Figure 2:**
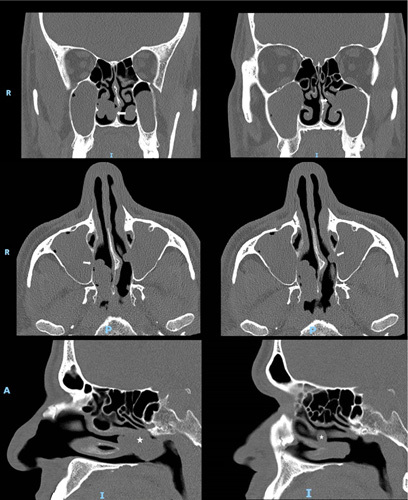
Computed tomography (CT) scan (coronal, axial, and sagittal views) of the paranasal sinuses depicting bilateral antrochoanal polyps (arrows, asterisks).

A 44-year-old male smoker (15 pack-years) with no significant surgical, medical, or family history was referred to our rhinology clinic with a 1-year history of right-sided nasal obstruction, rhinorrhea, postnasal drip, snoring, mouth breathing, sleep disturbances, occasional sternutation, and itching. He denied other sinonasal symptoms, such as epistaxis, hyposmia, headache, and facial pressure. Endoscopic examination of the right side revealed a solitary nasal polyp originating from the middle meatus occupying the NP and almost entirely obstructing the right choanae. The left side showed a much smaller polyp that barely extended from the middle meatus, with marked left nasal septal deviation at the same level. Bilateral hypertrophy of the inferior nasal turbinates (HIT) was also noted (Fig. 1). The remaining otorhinolaryngological examinations were unremarkable. Computed tomography (CT) of the paranasal sinuses without contrast showed a homogenously opacified soft-tissue density in the maxillary sinus bilaterally, with a marked extension of the mass lesion from the widened right maxillary ostium, occupying the nasal cavity and NP with no evidence of bone destruction or remodeling. Other associated CT findings included mucosal thickening in both sphenoid and ethmoid sinuses. Left nasal septal deviation with spur was observed near the left maxillary ostium including a right concha bullosa and HIT (Fig. 2). Consent for an endoscopic biopsy from both polyps under local anesthesia was granted, and histopathological evaluation revealed an inflammatory nasal polyp, ruling out malignancy.

Based on the recorded history, endoscopic nasal examination, and CT findings, a diagnosis of bilateral ACPs was made. The patient was then counseled for functional endoscopic sinus surgery (FESS), but he refused and decided to undergo medical treatment. The Arabic Sino-Nasal Outcome Test (SNOT)-22^16^ score was obtained to monitor symptom progression and was 73. The medical preparation consisted of a course of clarithromycin 500 mg twice daily for 10 days, mometasone furoate 50 µg/actuation aerosol spray twice daily, together with nasal rinses (0.9% sodium chloride irrigation solution) twice daily for 3 months. The patient was seen after the specified period, reporting significant improvements in his sinonasal symptoms with a SNOT-22 score of 34. The patient was satisfied with the results. He has been on regular 2–3 months follow-ups, with no reports of worsening symptoms till date.

## Discussion

ACP constitutes a minority of all benign nasal polyps, as it typically represents 4–6% of all nasal polyps in the general population; however, the rate peaks up to 33% among children^2^. As it grows in size, it fills the maxillary sinus to extend into the NP, thereby obstructing the choanae and causing patients to present with symptoms similar to many nasal disorders, most notably nasal obstruction, rhinorrhea, snoring, and mouth breathing^17^.

A comprehensive description of ACP was first provided by Killian in 1906. However, he could not trace its origin until 1909, when Ion Kubo established that ACP originated from the maxillary antrum^18^,^19^. Within the maxillary sinus, the medial and posterior walls have been reported to be the most common sites of origin, yet their exact anatomic origin remains inconsonant in the literature^20^. The accessory maxillary ostium acts as the exit passageway for ACP in 70% of cases^20^. This observation may elucidate why ACP grows and extends inferiorly and posteriorly into the NP.

**Figure 3:**
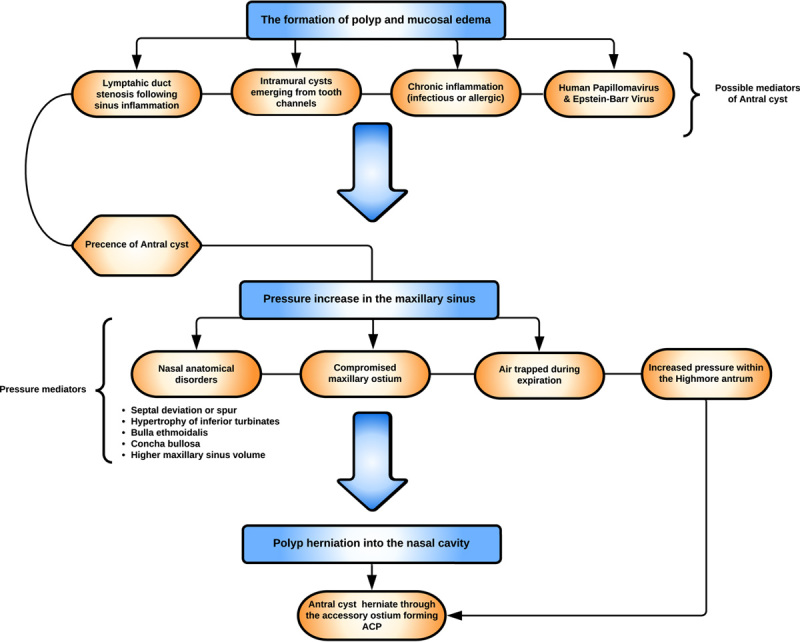
A simplified overview of the etiopathogenesis of an antrochoanal polyp^1^. ACP, antrochoanal polyp.

The quest behind comprehending the etiopathogenesis behind ACP and its origin remains controversial, with many reported hypotheses and observations. The most lingering theory to date is the presence of an antral cyst as the origin of ACP. A summary of the etiopathogenesis of ACP and possible mediators of antral cysts is depicted in Figure 3.

To substantiate this theory, a radiological study by Bidkar *et al*.^20^ reported consistent reductions in the thickness of the alveolar bone of the maxillary sinus suggesting the presence of a long-standing lesion, such as an antral cyst. The antral cyst herniates through the accessory maxillary ostium, creating an ACP because of the inflammatory changes, edema, and increased intrasinus pressure caused by the partial occlusion of the natural ostium, as hypothesized by Frosini *et al*.^1^. The rarity of bilateral ACPs can be explained by the lack of sufficient negative intranasal pressure on the contralateral side (because of the unilateral ACP occupying the NP) needed to pull the maxillary contents into the nasal cavity^8^. In our case, the left ACP was barely protruding from the maxillary ostium, with a left deviated nasal septum and spur at the same level, possibly hindering the ACP from extending further. This, in part, lends credence to the aforementioned hypothesis and contributes to it that anatomical nasal disorders, such as nasal septal deviation and spurs, may play a role in hampering ACP extension into the nasal cavity.

Moreover, diagnosing ACP is mainly clinical; however, certain diagnostic challenges remain because of the rarity and ambiguity of its clinical and radiological signs; therefore, meticulous endoscopic examination, a high index of suspicion, CT imaging, and postoperative histopathological findings are indispensable for an accurate diagnosis. In turn, this may spare patients from prolonged, unnecessary medical treatment and/or aggressive surgery.

The treatment of ACP is always surgical, with the FESS technique being the most preferred and effective procedure^17^. Optimal surgical results ensure that the ACP’s intramaxillary portion is entirely resected, as incomplete resection will lead to almost inevitable recurrence^1^. Regular follow-ups of at least 2 years postoperatively would be prudent to detect 95% of recurrent cases^21^.

In our case, the patient refused the surgical approach and was continued on the medical regimen while being monitored for the progression of his symptoms before and after treatment using the SNOT-22 score. After receiving adequate medical therapy that targeted his other sinonasal symptoms, including HIT, postnasal drip, allergic rhinitis, and quality of sleep, the patient’s symptoms and scores improved significantly. Nevertheless, the size of the ACP did not change. This is mainly because of the lack of inflammatory markers in ACP compared with that in chronic rhinosinusitis with nasal polyps^22^. Therefore, anti-inflammatory medications are futile in managing ACP; hence, strengthening the belief that ACP is a surgical rather than a medical issue.

To our knowledge, only 11 cases of bilateral ACP have been reported in the medical literature, and our case is the first to report symptom improvement after medical therapy. To further expand our understanding, we performed a literature review of all documented bilateral ACP cases by using relevant search terms (‘antraochoanal polyp’ and ‘bilateral antrochoanal polyp’) in both the title and abstract. Our search yielded 35 articles published in PubMed with no language restrictions, of which 11 were included in this study. Unilateral ACP cases and noncase reports were excluded. For analysis purposes, we also included our case. Data on the presenting symptoms of bilateral ACP, histological findings, treatment, complications, recurrence, and follow-up were collected for all cases. The cases included an equal number of male and female patients (*n*=6 vs. 6), with a mean age of 12–48 years. The predominant presenting symptoms were nasal obstruction and rhinorrhea, which is consistent with the symptoms of our case. Histological reports of all cases revealed the presence of benign inflammatory nasal polyps. Almost all patients were treated with complete surgical excision, except for our case. FESS is the most commonly used technique with no postoperative complications. No recurrence was reported during the mean follow-up period of 9.4 months (Table 1).

**Table 1:** Review of all reported cases of bilateral antrochoanal polyp in the literature

Case no.	Reference	Year of publication	Sex	Age	Presenting symptoms	Initial treatment	Surgery complications	Histology finding	Recurrence	Mean follow-up (months)
1.	Myatt and Cabrera^4^	1996	F	12	Nasal obstruction and rhinorrhea	FESS	Nonreported	Benign, inflammatory polyp	None	3
2.	Basu *et al*.^5^	2001	F	12	Nasal obstruction and globus sensation	Caldwell–Luc operation	Nonreported	Benign, inflammatory polyp	None	6
3.	Yilmaz *et al*.^6^	2007	F	24	Nasal obstruction, rhinorrhea, and globus sensation	FESS	Nonreported	Benign, inflammatory polyp	None	12
4.	Konstantinidis *et al*.^7^	2008	F	49	Nasal obstruction and rhinorrhea	FESS	Nonreported	Benign, inflammatory polyp	None	6
5.	Al-Qudah^8^	2011	F	11	Nasal obstruction, rhinorrhea, snoring, and mouth breathing	FESS	Nonreported	Benign, inflammatory polyp	None	18
6.	Sousa *et al*.^9^	2011	M	37	Nasal obstruction	Caldwell–Luc operation	Nonreported	Benign, inflammatory polyp	None	6
7.	Ozdek and Ozel^10^	2014	M	27	Nasal obstruction and rhinorrhea	FESS	Nonreported	Not mentioned	None	35
8.	Sabino *et al*.^11^	2014	M	48	Nasal obstruction, rhinorrhea, hyposmia, and sleeping disturbances	FESS	Nonreported	Benign, inflammatory polyp	None	4
9.	Oner *et al*.^12^	2015	M	20	Rhinorrhea, hyposmia, and headache	FESS	Nonreported	Benign, inflammatory polyp	None	Not mentioned
10.	Aksakal^13^	2018	M	11	Facial fullness and rhinorrhea	FESS	Nonreported	Benign, inflammatory polyp	None	8
11.	Iziki *et al*.^14^	2019	F	44	Nasal obstruction, hyposmia, and headache	FESS	Nonreported	Benign, inflammatory polyp	None	12
12.	Almarri et al.	2022	M	44	Nasal obstruction, rhinorrhea, snoring, mouth breathing, and allergic rhinitis	Medical treatment	NA	Benign, inflammatory polyp	NA	2–3

F, female; FESS, functional endoscopic sinus surgery; M, male; NA, not applicable.

This report is the first to demonstrate relief in symptoms of bilateral ACP with conservative treatment. This finding implies that a frugal approach through a trial of medical treatment might deem useful for patients who cannot undergo a major surgical procedure and/or tolerate general anesthesia.

## Conclusion

ACP is a benign solitary lesion that originates in the maxillary sinus and may occur in adults and children, with predominant symptoms manifesting as nasal obstruction and rhinorrhea. The etiology of ACP remains hypothetical, and its bilateral presentation is extremely rare due to either insufficient negative pressure on the contralateral side needed to draw the maxillary contents in the nasal cavity or the presence of an anatomical nasal disorder that may hamper the extension of ACP into the nasal cavity. Additionally, ACP harbor ambiguous clinical and radiological features, entailing a considerably high index of suspicion to provide a timely diagnosis, thereby sparing patients from extensive medical or surgical treatment. Successful ACP treatment depends on complete surgical excision to eliminate the risk of recurrence. Long-term postoperative monitoring for at least 2 years is advisable. A frugal approach through a trial of medical treatment may provide symptomatic relief in patients who are otherwise unfit for surgery and is therefore recommended.

## Ethical approval

The ethical committee approval was not required, given the article type is a case report. However, the written consent to publish the clinical data of the patients were given and is available to check by the handling editor if needed.

## Patent consent

Written informed consent was obtained from the patient for the publication of this case report and accompanying images. A copy of the written consent is available for review by the Editor-in-Chief of this journal on request.

## Patient perspective

Our patient responded positively to the medical management offered by the team as his condition improved drastically.

## Sources of funding

This case report did not receive any specific grant from funding agencies in the public, commercial, or not-for-profit sectors.

## Author contribution

F.K.A. and A.A.: conceived the study design; F.K.A.: performed the literature review; F.K.A. and A.M.A.: prepared the components of the case presentation; F.K.A.: drafted the manuscript, which was then reviewed by A.A. and A.M.A. All authors read and approved the final version of the manuscript.

## Conflicts of interest disclosure

The authors state that they have no conflicts of interest regarding the publication of this report.

## Research registration unique identifying number (UIN)

1. Name of the registry: not applicable.
2. Unique identifying number or registration ID: not applicable.
3. Hyperlink to your specific registration (must be publicly accessible and will be checked): not applicable.

## Guarantor

Firas K. Almarri, Department of Otorhinolaryngology – Head and Neck Surgery, Ad Diriyah Hospital, Riyadh Third Health Cluster, Ministry of Health, Riyadh, Saudi Arabia, Tel: +966548104118, E-mail: firasalmarri@gmail.com, ORCID: https://orcid.org/0000-0002-8981-411X

## Provenance and peer review

Not commissioned, externally peer-reviewed.
